# A Multicentre Study of *Shigella* Diarrhoea in Six Asian Countries: Disease Burden, Clinical Manifestations, and Microbiology

**DOI:** 10.1371/journal.pmed.0030353

**Published:** 2006-09-12

**Authors:** Lorenz von Seidlein, Deok Ryun Kim, Mohammad Ali, Hyejon Lee, XuanYi Wang, Vu Dinh Thiem, Do Gia Canh, Wanpen Chaicumpa, Magdarina D Agtini, Anowar Hossain, Zulfiqar A Bhutta, Carl Mason, Ornthipa Sethabutr, Kaisar Talukder, G. B Nair, Jacqueline L Deen, Karen Kotloff, John Clemens

**Affiliations:** 1 International Vaccine Institute, Seoul, Korea; 2 Fudan University, Shanghai, China; 3 National Institute of Hygiene and Epidemiology, Hanoi, Vietnam; 4 Faculty of Allied Health Sciences, Thammasat University, Rangsit Center, Patumthani, Thailand; 5 National Institute of Health Research and Development, Jakarta Indonesia, Ministry of Health, Jakarta, Indonesia; 6 Centre for Health and Population Research, Dhaka, Bangladesh; 7 Department of Paediatrics, The Aga Khan University, Karachi, Pakistan; 8 United States Armed Forces Research Institute for Medical Sciences, Bangkok, Thailand; 9 Centers for Vaccine Development, Baltimore, Maryland, United States of America; Institut Pasteur, France

## Abstract

**Background:**

The burden of shigellosis is greatest in resource-poor countries. Although this diarrheal disease has been thought to cause considerable morbidity and mortality in excess of 1,000,000 deaths globally per year, little recent data are available to guide intervention strategies in Asia. We conducted a prospective, population-based study in six Asian countries to gain a better understanding of the current disease burden, clinical manifestations, and microbiology of shigellosis in Asia.

**Methods and Findings:**

Over 600,000 persons of all ages residing in Bangladesh, China, Pakistan, Indonesia, Vietnam, and Thailand were included in the surveillance. *Shigella* was isolated from 2,927 (5%) of 56,958 diarrhoea episodes detected between 2000 and 2004. The overall incidence of treated shigellosis was 2.1 episodes per 1,000 residents per year in all ages and 13.2/1,000/y in children under 60 months old. Shigellosis incidence increased after age 40 years. S. flexneri was the most frequently isolated *Shigella* species (1,976/2,927 [68%]) in all sites except in Thailand, where S. sonnei was most frequently detected (124/146 [85%]). S. flexneri serotypes were highly heterogeneous in their distribution from site to site, and even from year to year. PCR detected *ipaH,* the gene encoding invasion plasmid antigen H in 33% of a sample of culture-negative stool specimens. The majority of S. flexneri isolates in each site were resistant to amoxicillin and cotrimoxazole. Ciprofloxacin-resistant S. flexneri isolates were identified in China (18/305 [6%]), Pakistan (8/242 [3%]), and Vietnam (5/282 [2%]).

**Conclusions:**

Shigella appears to be more ubiquitous in Asian impoverished populations than previously thought, and antibiotic-resistant strains of different species and serotypes have emerged. Focusing on prevention of shigellosis could exert an immediate benefit first by substantially reducing the overall diarrhoea burden in the region and second by preventing the spread of panresistant *Shigella* strains. The heterogeneous distribution of *Shigella* species and serotypes suggest that multivalent or cross-protective *Shigella* vaccines will be needed to prevent shigellosis in Asia.

## Abstract 2

A prospective, population-based study in six Asian countries showed that*Shigella* appears to be more ubiquitous in Asian impoverished populations than previously thought, and antibiotic-resistant strains have emerged.

## Editors' Summary

**Background.:**

Infections that cause diarrhea are a major public health problem in developing countries and other places where resources are scarce, particularly in young children. Although deaths from diarrhea have decreased considerably in recent decades, diarrheal illnesses continue to cause some 2.5 million deaths each year. *Shigella,* a group of rod-shaped bacteria closely related to those that normally live in the human intestine, is known to cause severe diarrhea in both developed and developing countries, but the global impact of *Shigella* infection (shigellosis) has not been well characterized. *Shigella* exists in more than 40 different varieties, an increasing number of cases have been found to be resistant to available antibiotics, and no vaccine is licensed except one oral vaccine in China.

**Why Was This Study Done?:**

The best information available on the impact of shigellosis has been based on historical estimates, which are subject to inaccuracy. More recent studies suggest that the older reports may have underestimated the impact of shigellosis. The authors of this study wanted to obtain more accurate, current estimates of the impact of shigellosis in developing countries.

In addition, immunity to one type of *Shigella* does not necessarily provide protection against other types. Therefore, in order to develop an effective vaccine, researchers would need to know which types of *Shigella* are causing illness in affected parts of the world. Accordingly, the authors of this study also wanted to investigate the specific types of *Shigella* (called “serotypes” because they can be distinguished using serum from immune individuals) involved in cases of diarrhea.

**What Did the Researchers Do and Find?:**

The researchers set up surveillance projects for diarrhea in six developing countries throughout Asia: at three rural or semirural sites (in China, Vietnam, and Thailand) and three urban slum sites (in Bangladesh, Pakistan, and Indonesia). They conducted information campaigns in each area to encourage residents to visit a participating clinic if they or their children developed diarrhea. Patients presenting with diarrhea were enrolled in the study and their medical findings were documented on standardized report forms. Stool or rectal swab specimens were obtained (with patient consent) and sent to laboratories to test for *Shigella*. When *Shigella* was identified, the bacteria was serotyped and tested for resistance to antibiotics. Because standard culture methods do not always detect *Shigella* when it is present, as a double-check, the researchers also tested some of the specimens for a type of DNA (called the *ipaH* gene) that serves as a molecular “footprint” of *Shigella*. Patients received treatment according to national guidelines.

The study involved approximately 600,000 participants over 1–3 years, and detected approximately 60,000 cases of diarrhea. Shigella was found in 5% of diarrhea episodes, meaning that two new cases of shigellosis occurred per 1,000 people (of all ages) per year. Rates were higher in children and in people over age 40. Among children less than 5 years old, there were 13 new cases per 1,000 children per year. Rates of shigellosis were higher in the Bangladesh site than in the China, Pakistan, and Indonesia sites, which in turn had higher rates than the Vietnam and Thailand sites.

In contrast to prior studies, no deaths were detected following episodes of shigellosis, and less than one-third of cases of shigellosis were associated with bloody diarrhea (dysentery).

The distribution of serotypes was found to differ from one site to another and within a given site over time. A high percentage of *Shigella* detected at all sites were resistant to two or more antibiotics. Testing for the *ipaH* gene was able to identify *Shigella* in half of patients with bloody diarrhea whose routine stool cultures did not reveal *Shigella*.

**What Do These Findings Mean?:**

This study found that shigellosis occurs in these Asian sites at a rate approximately 100 times higher than in industrialized countries. The finding that shigellosis frequently occurs in the absence of bloody stool means that government data collections using dysentery as part of the case definition can be expected to miss the majority of shigellosis cases. Also, the increased rate of shigellosis above age 40 shows that older people share significantly in the burden (and most likely the transmission) of shigellosis.

The generally benign clinical course of *Shigella*-associated diarrhea calls into question the priority that this disease should receive in global vaccine development efforts, especially given the technological challenges posed by the complex and variable distribution of serotypes. Nonetheless, the emergence of multidrug-resistant strains clearly remains a threat, and raises the perennial issue of improved sanitation, rather than new antibiotics, as a long-term solution to the plethora of water-borne illnesses that disproportionately affect developing countries.

**Additional Information:**

Please access these Web sites via the online version of this summary at http://dx.doi.org/10.1371/journal.pmed.0030353.

- World Health Organization topic page on diarrhea
- Centers for Disease Control and Prevention: Shigellosis
- Wikipedia entry on Shigella (note: Wikipedia is a free Internet encyclopedia that anyone can edit)

## Introduction

The burden of shigellosis is greatest in resource-poor countries where the disease may cause as many as 167 million episodes of diarrhea and over a million deaths annually [1]. Previously efficacious drugs such as sulphonamides, tetracycline, ampicillin, and trimethoprim-sulfamethoxazole have become largely ineffective against prevalent *Shigella* strains. The recently reported emergence of ciprofloxacin resistance further narrows the choice of effective antimicrobials [2–4].

S. flexneri species are known to have 15 serotypes and subtypes, S. dysenteriae 13 serotypes, S. boydii 18 serotypes, and S. sonnei a single serotype. Several vaccine candidates are under development [5–10], but these may need to be tailored according to prevalent species and serotypes, since only type-specific immunity has been demonstrated in humans [11–13] and cross-serotype protection is controversial [14]. A detailed understanding of the epidemiology of shigellosis is essential for the rational development of potential vaccine candidates to control shigellosis.

Because of the lack of recent, reliable data on shigellosis, especially from nonindustrialized countries, we conducted a prospective, multicentre, population-based study of the burden and patterns of shigellosis in six developing countries of Asia.

## Methods

To allow pooling of data and comparisons across study sites, standardized epidemiologic, clinical, and laboratory methods were employed. Prior to the project start, methods were agreed upon by the principal investigators during workshops. The studies were monitored by epidemiologic and laboratory coordinators during regular visits to the study sites. A full description of healthcare systems and healthcare utilization patterns in the study sites [15–20] as well as epidemiologic and microbiologic methodology in the study sites in Indonesia [21], China [22], and Thailand [23] have been presented elsewhere.

### Study Sites and Population

Surveillance was conducted at study sites in six developing countries of Asia: three rural or semirural areas (China, Vietnam, and Thailand) and three urban slums (Bangladesh, Pakistan, and Indonesia) (Table 1). The population size in each study site was estimated through existing, recent census data (China, Thailand, Indonesia) or a census specially conducted for the purpose of the study (Bangladesh, Pakistan, Vietnam).

**Table 1:**
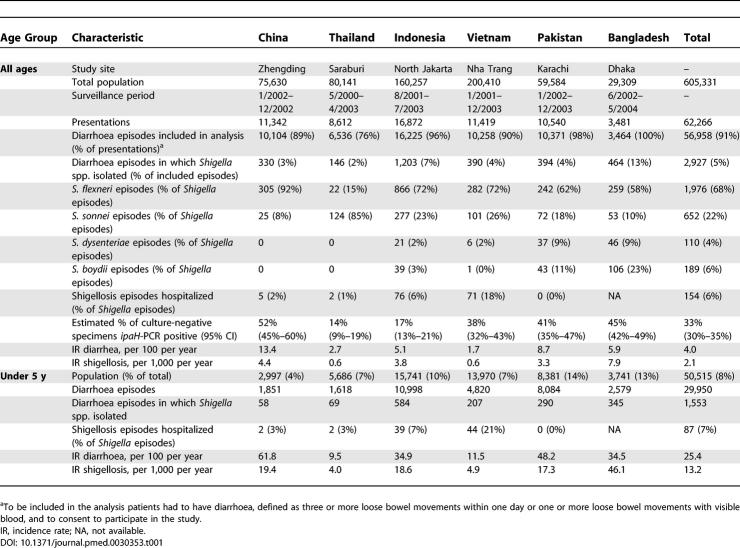
Study Population, Disease Episodes, and Incidence at Six Study Sites

The research site in China was in Hebei Province, approximately 270 km south of Beijing. The catchment area consisted of 29 villages in four rural townships in Zhengding County with a total population of 75,630 in 2000, of which 2,997 (4%) were children under age 60 mo. About 80% of the study population were agricultural workers. Surveillance was conducted in 101 village clinics and four township hospitals.

The Thailand study area was in Kaengkhoi District, Saraburi Province, approximately 100 km north of Bangkok. The area includes a small city surrounded by rural villages that depend on agriculture for income. In 2001 80,141 people lived in the catchment area, including 5,686 (7%) children under age 60 mo. Surveillance was conducted in 20 community health centres and the district hospital.

In Indonesia, two adjacent districts *(kecamatans)* in North Jakarta, Tanjung Priok, and Koja, formed the study area. Many homes are temporary structures without running water, and more than one-third of households have no access to tap water. The area is prone to flooding during the rainy season. The main occupations include harbour labour, small business, and clerical work. In 2000 the population in the catchment area was 160,257, of whom 15,741 (10%) were under age 60 mo. Surveillance was conducted in eight public health care centres *(puskesmas)* and two hospitals (the Infectious Disease Hospital and Koja Hospital).

In Vietnam, the study was conducted in the coastal city of Nha Trang, the provincial capital of Khanh Hoa Province in the central part of the country. The population in the catchment area was 200,410, of which 13,970 (7%) were under age 60 mo. This population is mostly employed in fisheries, agriculture, and tourism. Surveillance was conducted in community health centres of 16 communes, in four polyclinics, and in the general hospital.

In Pakistan the research site included four low-income communities. Rehri Goth, a suburban fishing village, and Sherpao Colony, where most people earn a living as labourers, are both southeast of Karachi. Hijrat Colony and Sultanabad, two contiguous urban slums, are near the business centre and port of Karachi. The catchment area in 2002 had a population of 59,584, of which 8,381 (14%) were children under age 60 mo. In each of the four communities a treatment centre was established for the purpose of surveillance.

In Bangladesh the study catchment area was in Kamlapur, a densely populated settlement of rural immigrants in the southeastern sector of Dhaka city. The population in the catchment area was 29,309, of which 3,741 (13%) were children under age 60 mo. The rapid growth of the settlement has resulted in a mixture of permanent structures and temporary squatter dwellings. The most common occupations are trading, clerical work, and rickshaw puller. One central treatment centre was used for surveillance.

### Project Design

Before and during surveillance, information campaigns were conducted to encourage all residents in each of the catchment areas to visit a participating health care centre and provider for all diarrhoea episodes. Individuals of all ages presenting with diarrhoea or dysentery were enrolled in the study. The clinical history and physical findings of each patient were documented on standardized case report forms.

A rectal swab or bulk stool was obtained from each patient who provided verbal informed consent. The rectal or bulk stool swabs were inserted into 1.5 ml of buffered glycerol saline or Cary Blair medium, refrigerated until collection by a courier, transported in a cool box to the central laboratory by motorcycle or car, and plated on the day of collection. Participants received treatment according to national guidelines.

### Definitions

Diarrhoea was defined as three or more loose bowel movements during a 24-h period, dysentery as one or more loose bowel movements with visible blood. A diarrhoeal episode was defined as new if the diarrhoea definition was met after three or more days free of diarrhoea or dysentery [24]. A shigellosis episode was defined as a diarrhea episode during which a faecal specimen was obtained from which any *Shigella* species was isolated. An episode of persistent diarrhea was defined as an episode of diarrhea lasting for 14 d or more.

### Laboratory Procedures

The swabs were inoculated in MacConkey agar and *Salmonella-Shigella* agar. After overnight incubation at 37 °C, the MacConkey agar and *Salmonella-Shigella* agar plates were checked for nonlactose-fermenting colonies. Three colonies characteristically resembling *Shigella* were differentiated from other nonlactose-fermenting enteropathogens by inoculating into Kligler's iron agar for typical reaction, mannitol fermentation, citrate utilization, urease and indole production, and lysine decarboxylation. After incubation for 18–24 h at 37 °C, the test media were read for characteristic *Shigella* reactions.

Serologic identification was performed by slide agglutination with polyvalent somatic (O) antigen grouping sera, followed by testing with monovalent antisera for specific serotype identification (antisera from Denka Seiken, Japan, were used in all sites). In cases where no agglutination occurred with live bacteria, the test was repeated with boiled suspensions of bacteria. S. flexneri isolates that were not typeable with commercial antisera were evaluated at Centre for Health and Population Research, Dhaka, Bangladesh using a panel of monoclonal antibodies specific for S. flexneri group and type factor antigen [25–28].

Antimicrobial susceptibility testing of *Shigella* isolates against ampicillin, cotrimoxazole, nalidixic acid, and ciprofloxacin was performed by disk diffusion following standardized National Committee for Clinical Laboratory Standards methods. Specimens were processed in local laboratories (Bangladesh: Centre for Health and Population Research, Dhaka; China: Preventive Health Laboratory, Zhengding; Pakistan: Aga Khan University, Karachi; Indonesia: United States Navy Medical Research Unit 2, Jakarta; Vietnam: Institute Pasteur, Nha Trang; and Thailand: Kaengkhoi Hospital, Saraburi).

The species, serotypes, subtypes, and antimicrobial resistance patterns of a sample of *Shigella* strains from the study sites in Vietnam, Bangladesh, and Pakistan were confirmed at the United States Armed Forces Research Institute for Medical Sciences, Bangkok during the initial months of each study. All *Shigella* isolates from the site in Thailand were confirmed at the World Health Organization National *Salmonella* and *Shigella* Centre, Ministry of Public Health, Nonthaburi, and all isolates from the study site in China were confirmed in Fudan University, Shanghai.

### Real-Time PCR

PCR can be used to amplify the gene coding for the invasion plasmid antigen H *(ipaH),* a gene nearly exclusively derived from the four Shigella spp. in Asia. The only alternative source of *ipaH* are enteroinvasive *E. coli,* an organism that is exceedingly rare in the Asian region [29].

Studies using *ipaH*-based PCR have been published from several Asian countries, including Thailand, Bangladesh, and more recently, India [30–32]. These studies suggest that *ipaH* can be detected in a large percentage of patients with diarrhoea who are culture-negative for *Shigella*. It is likely that *ipaH* detection rates differ not only between stool specimens from culture-positive and culture-negative diarrhoea patients but also between age groups and patients with dysentery and patients with diarrhoea without visible blood. Stool specimens from diarrhoea patients were therefore classified into eight categories as follows: by culture status (Shigella spp. isolated yes/no), presentation (dysentery/nondysentery), and age group (under 60 mo, 60 mo and older). Because it was not feasible to test all culture-negative stool specimens, a sample of specimens was selected from each of the above categories at each site.

The sample size required to detect a 95% prevalence of *ipaH* within a 95% confidence interval (CI) of 86%–99% was 60, and the sample size required to detect a 35% prevalence of *Shigella* DNA within a 95% CI of 26%–43% was 125. If more specimens had been collected than were required for testing, samples were randomly selected using a computer algorithm. 560 specimens each from Vietnam, Pakistan, and Indonesia were tested. For logistic reasons, a smaller sample was tested from China (*n* = 337) and Thailand (*n* = 320). The Bangladesh site provided 980 specimens from diarrhoea patients, of which 39 were culture-confirmed shigellosis cases.

A real-time PCR targeting *ipaH* was employed to detect *Shigella* DNA in faecal specimens [33]. Briefly, the fluorogenic probe (FAM-CGC CTT TCC GAT ACC GTC TCT GCA-TAMRA) and its flanking primer pair (forward primer *ipaH* U1, 5′- CCT TTT CCG CGT TCC TTG A-3′; reverse primer *ipaH* L1, 5′- CGG AAT CCG GAG GTA TTG C-3′) were designed on the basis of *ipaH* gene sequences.

For real-time PCR detection, faecal swabs were washed in 0.8 ml of PBS, of which 0.5 ml was pipetted into 1.5 ml microcentrifuge tubes. The tubes were incubated in boiling water for 30 minutes to lyse bacterial cells. The lysate was subjected to centrifugation at 10,000 rpm for 1 min. The lysate was either used directly for real-time PCR or stored at −70 °C. The working cocktail for the detection contained 1 μl of DNA template, 1× TaqMan buffer A (Applied Biosystems, Foster City, California, United States), 2 mM MgCl_2_, 100 nM each of dNTPs, 200 nM of primers (*ipaH*-U1 and *ipaH*-L1), 40 nM of fluorogenic probe, *ipaH*-P1 (TET-labelled), and 1.25 units of AmpliTaq Gold (Applied Biosystems) in 25 μl of total reaction volume. The TaqMan assays were conducted using an ABI 7700 Detection System (Applied Biosystems). The amplification profile consisted of heat activation at 95 °C for 10 min; 40 cycles of denaturation at 95 °C for 30 s; and annealing, extension, and fluorogenic probe hybridization at 60 °C for 1 min. The assay was considered positive when the number of cycles to detection was 38 or less. Real-time PCR-negative samples found to contain inhibitors were further purified using Qiagen Stool Kit (Qiagen, Valencia, California, United States). All PCR assays were conducted at the USAFRIMS, Bangkok, Thailand, and technicians were blinded as to the culture status and clinical presentation of the participants from which the faecal specimens were obtained.

### Data Management and Analysis

Data were double entered into a custom data entry program (FoxPro, Microsoft, Redmond, Washington, United States) with error- and consistency-check programs. Annualised incidence per thousand population was calculated by using age-specific denominators from the baseline census at each site. The observation periods ranged from 12 to 36 mo at each site, and we assumed that each person residing in the study area at the time of the census contributed the respective months to the denominator. The number of all age-specific disease episodes including repeat episodes was used as the numerator. We calculated 95% CIs for incidence rates by the Wilson score method [34]. For intergroup comparisons, we used Chi-square tests for comparison of categorical variables. For the analysis of continuous variables, Student's t-test was used for normally distributed and Wilcoxon rank sum, and Kruskal-Wallis test for non-normally distributed data. Rate ratios were used to detect statistically significant differences between incidence rates.

Because the presentations varied by age, species, and site, adjusted logistic regression models were used to assess whether symptoms were more frequent in shigellosis patients compared to diarrhoea patients from whom no *Shigella* species were isolated. The models took the presence of a sign or symptom in each analyzed diarrhoea episode as the dependent variable and fitted the shigellosis status (shigellosis or a diarrhoea episode during which no Shigella spp. could be isolated) and biologically plausible potential confounders (age and site) as independent variables. Interactions were tested to estimate the effect of age, site, and *Shigella* species on the frequency of the presenting signs and symptoms. In a secondary analysis, the shigellosis status was replaced as the independent variable with individual *Shigella* species to evaluate the association between individual species and presentations. The analysis of factors associated with persistent diarrhoea was conducted in an analog fashion.

Coefficients of independent variables in the models were exponentiated to estimate the odds ratio of symptoms associated with shigellosis status. Standard errors for the coefficients were used to estimate two-tailed *p*-values and associated 95% CIs for the odds ratios. The disproportionate sampling of specimens for PCR analysis was accounted for in the analysis which based on weighted averages. A *p*-value of less than 0.05 (two-tailed) was considered significant. Stata/SE 8.0 (Stata Corporation, College Station, Texas, United States) was used for the analysis.

### Ethics and Informed Consent

After the project's purpose was explained, patients—or in the case of minors, their parents or guardians—gave verbal consent prior to participation in the study. Participation consisted of providing faecal specimens and the information required to complete the case report forms. All studies were approved by each site's local ethics review committees. International approval for all studies, except the study in Bangladesh, was obtained from the Secretariat Committee for Research Involving Human Subjects, WHO, Geneva, Switzerland. The study in Bangladesh was approved by the Bangladeshi Government and by the ICDDR,B ethics review board, which has international membership.

## Results

**Figure 1:**
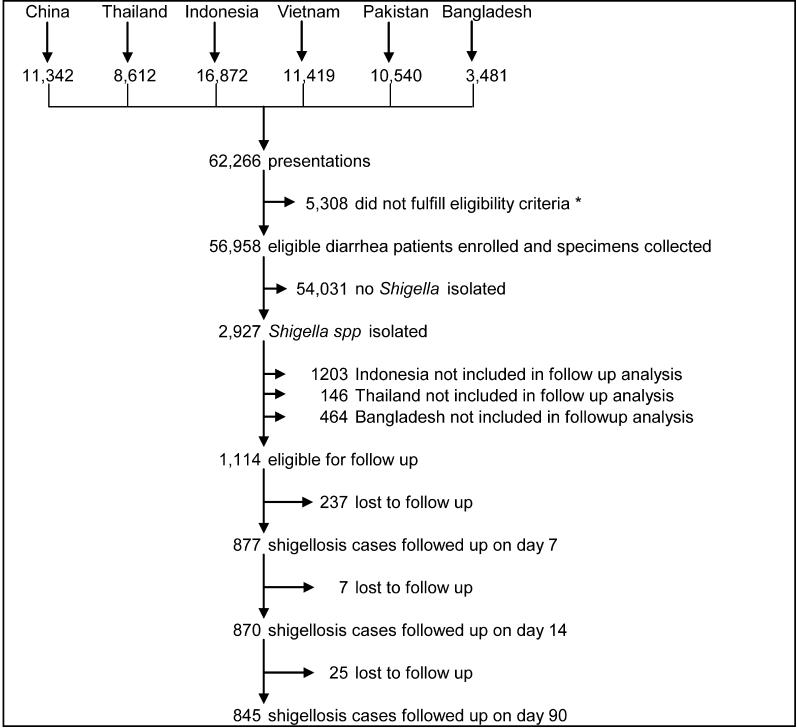
Assembly of Cases * Eligibility criteria: three or more bowel movements per 24 h or at least one loose stool with blood, and consent from patient or parent/guardian.

In the six sites, a total of 605,331 people were studied between 1 and 3 years, resulting in 1,415,583 person-years of shigellosis surveillance (Table 1). The project detected 62,266 diarrhoea episodes, of which 56,958 (91%) fulfilled the eligibility criteria, which consisted of diarrhoea for 1 d or more and patient consent to participate in the study (Figure 1).

### Incidence

**Figure 2:**
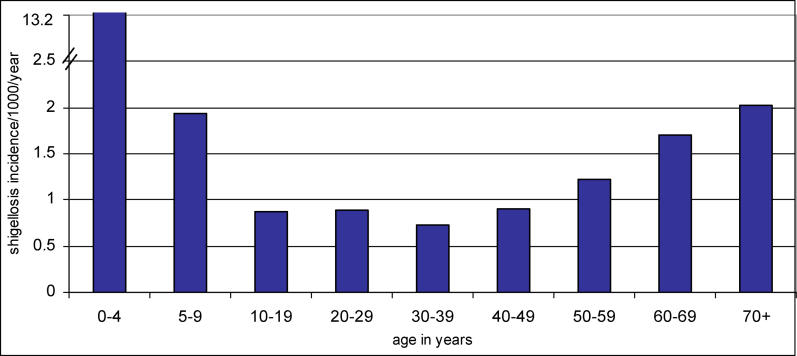
Overall Shigellosis Incidence by Age Group at Study Sites in Six Asian Countries Note: Shigellosis incidence in the age group 0–4 y is 13.2/1,000/y.

The overall diarrhoea incidence was 40 episodes per 1,000 patients per year in all age groups and 254/1,000/y among those under age 60 mo. *Shigella* was isolated from 2,927/56,958 (5%) of diarrhoea episodes*.* The overall incidence of treated shigellosis was 2.1 episodes/1,000/y in all ages and 13.2/1,000/y in children under age 60 mo. The shigellosis incidence increased after age 40 y (test for trend, *p* < 0.001; Figure 2). The shigellosis rates of the overall population as well as in children under 5 y in the site in Bangladesh were statistically significantly higher than the shigellosis rates in China, Pakistan, and Indonesia (*p* < 0.001), which in turn were significantly higher than those in the two countries with the lowest shigellosis rates, Vietnam and Thailand (*p* < 0.001; Table 1).

### Clinical Presentations of Shigellosis Episodes

**Figure 3:**
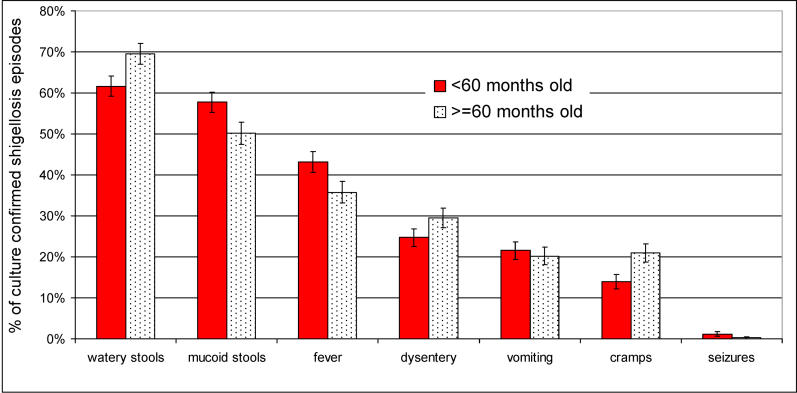
Clinical Presentation of Shigellosis Episodes A history of more than one clinical sign and symptom during a single episode is possible.

Culture-confirmed shigellosis cases frequently reported more than one clinical sign or symptom, ranging from watery stools through mucoid stools to dysentery. During 1,807 (65%) of 2,766 culture-confirmed shigellosis episodes, watery diarrhoea was reported; in 1,518 (54%) of 2,802 episodes patients reported mucoid stools; and 790 (27%) of 2,925 episodes patients reported dysentery (Figure 3). In multiple logistic regression models adjusted for study site and age of the patient four clinical signs and symptoms correlated positively with the detection of *Shigella* spp in stool specimens: dysentery, mucoid diarrhoea, fever, and abdominal cramps (Table 2). In contrast, watery diarrhoea and vomiting were significantly more frequently reported during diarrhoea episodes from which no Shigella spp. was isolated. The percentage of shigellosis patients admitted for hospital care varied between the sites. None of 394 shigellosis patients in Pakistan was admitted, in contrast to 71 of 390 (18%) shigellosis cases in Vietnam (Table 1).

**Table 2:**
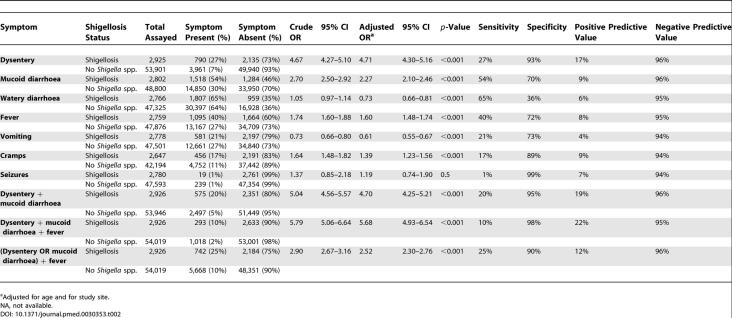
A Comparison of Clinical Presentation among Patients from whom Shigella spp. Was and Was Not Isolated

### Differences in the Age of Shigellosis Patients between Study Sites

The median age of shigellosis patients in Bangladesh and Pakistan was 2 y; in Vietnam was 4 y; and in Indonesia and Thailand 5 y. China was a outlier with a median age of 32 y (Kruskal-Wallis test; 5 degrees of freedom; *p* < 0.001). By age 2 y, 37% and 38% of shigellosis patients in Bangladesh and Pakistan, respectively, had acquired the infection; in Indonesia and Vietnam 30% and 28%, respectively; in Thailand 12%; and in China 5% (Chi-square test; 5 degrees of freedom; *p* < 0.001).

### Outcome of Shigellosis Episodes

Of 1,114 eligible shigellosis patients, 870 (18%) were followed for 14 d or longer, and 845 (76%) were followed for 90 d in the study sites in China, Vietnam, and Pakistan. Of the 870 (18%) shigellosis patients who were followed for at least 14 d, 153 (18%) reported that the total diarrhoea episode lasted for 14 d or more (Table 3); 91 (11%) stated that diarrhoea was still present 14 d after presentation. Of 845 patients with culture-confirmed shigellosis, 21 (3%) reported medical events such as pneumonia during the 90 d follow-up period. No deaths were detected following shigellosis episodes. Five clinical signs and symptoms at the time of presentation were statistically significantly associated with persistent diarrhoea in adjusted regression models age, fever, mucoid diarrhoea, vomiting, and abdominal cramps (Table 4). No statistically significant association was detected between *Shigella* species, antimicrobial resistance, and persistent diarrhoea.

**Table 3:**
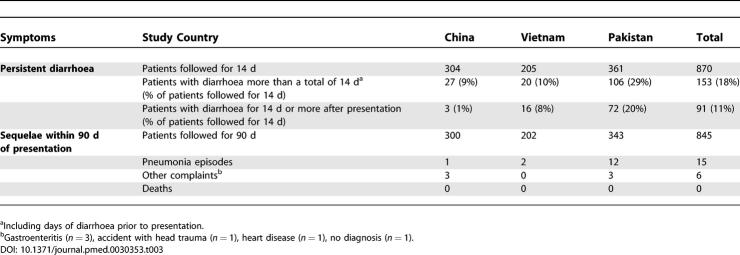
Persistent Diarrhoea and Other Sequelae of 870 Shigellosis Episodes in China, Vietnam, and Pakistan

**Table 4:**
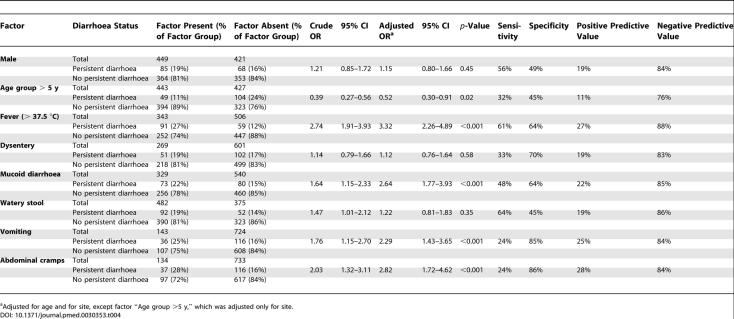
Factors Associated with Persistent Diarrhoea in Shigellosis Patients

### Microbiology

**Figure 4:**
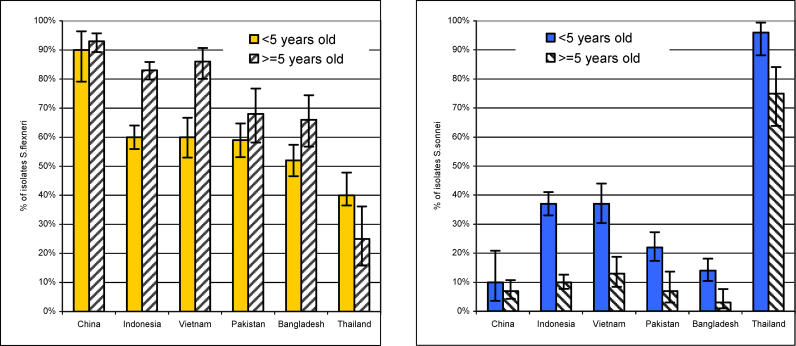
The Relative Distribution of S. flexneri and *S. sonnei* at Study Sites in Six Asian Countries S. flexneri (left bar graph) was more frequently isolated from diarrhea patients 5 y and older (*p* < 0.0001). In contrast, S. sonnei (right bar graph) was more frequently isolated from children under 5 y of age (*p* < 0.0001).

S. flexneri was the most frequently isolated species (1,976 [68%] of 2,927) at all sites except in Thailand, where S. sonnei was most common (124 [85%] of 146; *p* < 0.001; Figure 4). S. boydii was infrequently isolated, except in Bangladesh where it was the second most-common species, accounting for 23% (106 of 464) of shigellosis episodes*.* In all, 110 (4%) S. dysenteriae serotypes were isolated but none was S. dysenteriae type 1. In each of the six study sites S. flexneri was significantly more frequently isolated from patients ages 60 mo or older than from children under age 60 mo (Figure 4, left bar graph; *p* < 0.0001). In contrast, S. sonnei was more frequently isolated from children under age 60 mo than from patients age 60 mo or older (Figure 4, right bar graph; *p* < 0.0001). Dysentery and cramps were significantly more frequently reported by patients with diarrhoea from whom S. flexneri was isolated (Table 5).

**Table 5:**
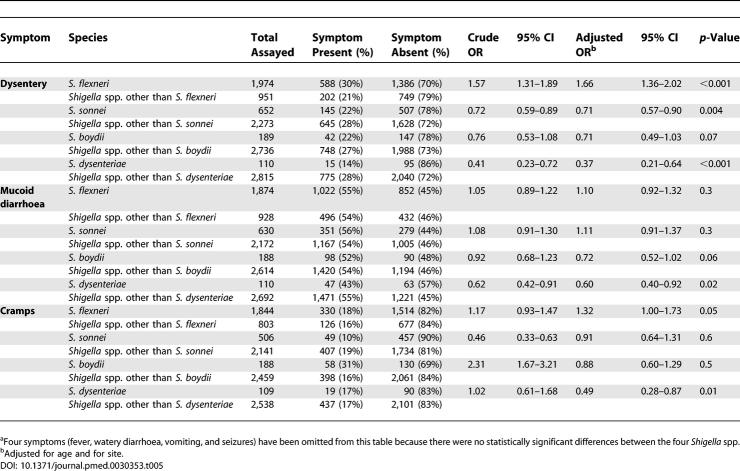
Clinical Presentation^a^ of Each Shigella spp. Compared with All Other Shigella spp. Isolated

The eight most frequently isolated S. flexneri serotypes (in order of prevalence 2a, 3a, 1a, 1b, 2b, 1c, 6, x) were responsible for 90% of all S. flexneri episodes in all five study sites where S. flexneri is the dominant *Shigella* species (Table 6). There were statistically significant differences in serotype prevalence of S. flexneri isolates between study sites (Chi square; 40 degrees of freedom; *p* < 0.001).

**Table 6:**
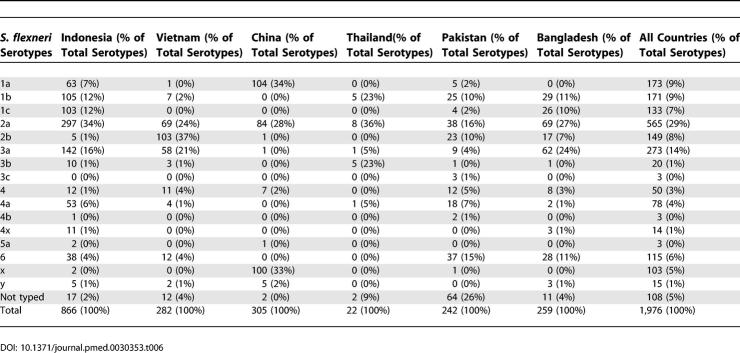
The S. flexneri Serotype Distribution in Six Study Sites

In Indonesia, Bangladesh, and Pakistan the S. flexneri serotype distribution was compared from one year to the next and in Thailand and Vietnam over 3 y. Statistically significant shifts in the in the relative proportions of S. flexneri serotypes were seen in each site (*p* < 0.001), with the exception of the site in Thailand, where over a 3 y period only 22 S. flexneri strains were isolated and no statistically significant shifts in the distribution of serotypes were observed.

### Antimicrobial Resistance

A high percentage of *Shigella* strains were resistant to ampicillin and cotrimoxazole at all sites (Table 7). The highest percentage of ampicillin-resistant isolates was found in S. flexneri (84%), followed by S. dysenteriae (37%), S. boydii (25%), and *S. sonnei* (10%; *p* < 0.001). In contrast, the highest percentage of cotrimoxazole-resistant specimens were S. sonnei (92%), followed by S. flexneri (76%), S. dysenteriae (62%), and S. boydii (49%; *p* < 0.001). Resistance to nalidixic acid varied widely, from 100% of S. flexneri and 98% of S. sonnei in China, through 75% of S. sonnei and 48% of S. flexneri in Bangladesh, to little or no resistance at the other sites. Ciprofloxacin-resistant S. flexneri isolates were identified in China (18 [6%] of 305), Pakistan (8 [3%] of 242), and Vietnam (5 [2%] of 282). Of the 1,653 ampicillin-resistant and the 1,490 cotrimoxazole-resistant S. flexneri isolates, 1,322 were cross-resistant to ampicillin and cotrimoxazole. Eighteen isolates were resistant to all four tested antimicrobials.

**Table 7:**
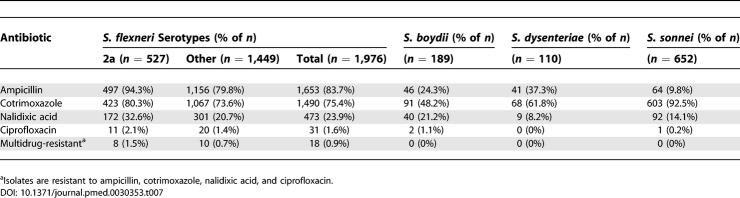
Antimicrobial Resistance Pattern for Four *Shigella* Species

### Detection of *ipaH* using Real-Time PCR

Of 427 *Shigella* culture-positive specimens, *ipaH* was detected in 385 (90%). In 1,124 culture-negative patients who reported dysentery, 564 (50%) had *ipaH* in the stool, in contrast to 673 of 1767 (38%) patients with nonbloody diarrhoea (*p* = 0.0001). The highest percentage of culture-negative, *ipaH*-positive specimens was detected in the two countries with the highest shigellosis incidence, China (52%; 95% CI, 45%–60%), and Bangladesh (45%; 95% CI, 42%–49%). In Thailand, one of the sites with the lowest shigellosis incidence rate, *ipaH* was detected in 14% (95% CI, 9%–19%) of stool samples (Table 1). In Vietnam, another country with relatively low shigellosis incidence, *ipaH* was detected in 38% (95% CI, 32%–43%) of stool samples.

**Figure 5:**
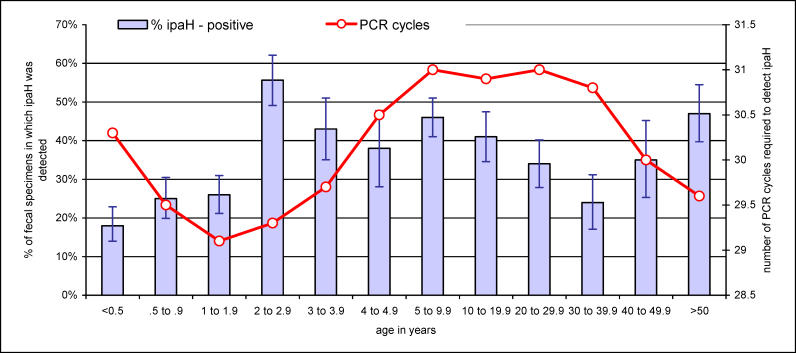
Relation of Proportion of *ipaH*-Positive Faecal Specimens and PCR Cycle Number to Age The percentage of *Shigella* culture-negative stool specimens from diarrhea patients in which *ipaH* was detected and the mean PCR cycle number required to detect *ipaH* by patient age suggests that children between ages 2 and 4 y and adults over age 40 y with diarrhoea are most likely to have *ipaH* in their stool specimens and the bacterial load is likely to be highest in these two age groups.

*IpaH* detection was lowest in children under age 6 mo, peaked between the second and tenth year of life, after which the percentage of *ipaH*-positive specimens declined until age 40 y, at which time the percentage of positive specimens increased again (Figure 5). Due to the semiquantitative nature of real-time PCR it is possible to compare the relative bacterial load between age groups, which is inversely related to the number of cycles required to detect *ipaH* (Figure 5). The bacterial load was relatively low during the first year of life and peaked during the second year of life. Between 5 and 40 years of life the bacterial load was relatively low, but it increased again after age 40 y.

## Discussion

This first multicentre shigellosis surveillance study found that shigellosis is more ubiquitous than previously thought. At six Asian study sites the overall culture-confirmed shigellosis annual incidence was 13.2 per 1,000 children under age 5 y and 2.1/1,000 in all ages. The shigellosis incidence in the study sites is approximately 100-fold higher than in industrialized countries. In the US in 1999 and the Netherlands 1996–2000 the estimated incidence shigellosis incidence in all ages was 3.7 and 3.2, respectively, per 100,000 per year [35,36]. Shigellosis incidence at our Asian study sites is in the same range as in Chile, where Prado and coworkers reported 9.0–12.6 shigellosis episodes per 100 children aged 12–47 mo in a semirural area between 1994 and 98 [37].

Although considerable shigellosis burden was detected, the actual burden caused by shigellosis was underestimated for two reasons. First, in using passive surveillance for case detection, we depended on the healthcare-seeking behaviour of individual patients. Studies of the care-seeking behaviour conducted in the context of the shigellosis surveillance studies in each site found significant differences in treatment uptake for diarrhoea and dysentery not only between sites but also within sites between adults and children and between patients presenting with diarrhoea and dysentery [15–20]. Shigellosis patients who treated themselves or sought healthcare from providers outside the surveillance system could not be captured. Active surveillance would have provided a more complete detection of all diarrhoea episodes at the risk of capturing trivial episodes that do not require medical care. Data collected in the mid-1980s in a poor, periurban community in Santiago, Chile, indicated that among children under one year of age, 88% of episodes of diarrhoea were mild cases that did not require health care but were detected by active household surveillance [13]. Studies using alternative designs such as active case detection could provide a more complete understanding of the shigellosis burden in the study sites.

Second, Shigella spp. are highly fastidious organisms that die rapidly in an unsuitable environment, including the unavoidable temperature fluctuations encountered during transport. Therefore a sample of culture-negative stool specimens from each site was subjected to PCR analysis, which has been found to highly sensitive and specific for Shigella spp. in Asia [33]. Evidence of *Shigella* DNA was detected in one-third of culture-negative stool specimens. The proportion of PCR-positive stool specimens correlated with age groups, thus lending support to the PCR findings. Presently it is unknown whether the detection of genetic material related to *Shigella* indicates disease, colonisation, or asymptomatic carriage. The percentage of PCR-positive specimens should therefore be viewed as upper limits of diarrhoeal disease potentially caused by Shigella spp.

Besides these limitations we found two additional explanations for earlier underestimates of the shigellosis burden. We found that less than one-third of culture-proven shigellosis episodes presented with dysentery. Clinical case definitions that include only patients with a history of dysentery, frequently used in government data collections, miss more than two-thirds of shigellosis cases. Lastly, in contrast to many other enteric infections, shigellosis is clearly not confined to childhood. On the contrary, the incidence of shigellosis not only increased steadily after age 40 y, but the bacterial load of shigellosis patients increased after age 40 y, suggesting that older people as well as very young children shed the highest bacterial load and may contribute disproportionally to the transmission of shigellosis. Based on these observations, we hypothesize that shigellosis is responsible for a larger proportion of the diarrhea burden in Asia than was previously inferred from culture results or clinical diagnoses.

Equally surprising was the benign clinical course of the shigellosis episodes. No deaths were detected and only 21/845 (3%) of patients reported medical events during follow-up. Persistent diarrhea was seen in 18% of patients following shigellosis episodes; however, the clinical importance of these persistent episodes is not clear. Earlier reports from the Asian region have stressed the potential severity and poor outcome of shigellosis episodes [38–40]. Several explanations for the unexpectedly low morbidity and mortality following shigellosis have been considered. First, by consenting to participate in the study, patients were assured to receive adequate treatment. Second, the Shigella strains may have changed during the decade since earlier reports on high shigellosis morbidity and mortality appeared. The absence of S. dysenteriae type 1, the only *Shigella* species with chromosomal genes encoding the 70-kDa heterodimeric protein known as Shiga toxin, supports this suggestion [41]. Third, earlier studies reporting high morbidity and mortality were hospital-based. As only the most severe shigellosis cases are admitted, the study population is likely to differ from outpatients enrolled in our study. Overall in our study, 6% of shigellosis patients were admitted, with large differences in hospitalization rates between sites. The range of hospitalization rates is perhaps best explained by differences in hospitalization policies between countries; for example, Vietnam has a very low threshold for triggering admissions compared to Pakistan. Finally, the host characteristics have changed over the last decade. While severe malnutrition in childhood remains a problem in the region, the prevalence of malnutrition has declined over the last ten years with the steady increase of economic markers. The widespread increase in income has contributed to the easy access to antibiotics in each of the study sites. In general, patients may have become less vulnerable to severe disease due to the availability of better nutrition and early self-treatment with antibiotics.

The over-the-counter sale of antibiotics without prescription enjoys popularity in all our study sites and may be responsible for the emergence of antibiotic resistance. The project confirmed that ampicillin and cotrimoxazole no longer have a place in the treatment of shigellosis. Nalidixic acid was recommended by the WHO as the first-line treatment against shigellosis until 2004, when it was replaced by ciprofloxacin [42]. Complete resistance to nalidixic acid in China and high levels of resistance in Bangladesh have clearly reduced the usefulness of this drug, at least in these two countries. Already 6% of S. flexneri isolates in China are resistant to ciprofloxacin. The emergence of multidrug-resistant *Shigella* isolates could reverse the benign course of shigellosis episodes observed in this study.

The prevention of shigellosis could exert an immediate benefit by substantially reducing the diarrhea burden in the region and by preventing the spread of panresistant *Shigella* strains. Safe water supplies and adequate sanitation combined with improved hygiene are likely to reduce the shigellosis burden in the future. Steady economic growth is likely to overcome the barriers currently obstructing improvements in infrastructure for underserved populations. But such progress can take decades and will provide little relief to unstable and mobile populations. In this context a safe and affordable vaccine to protect against shigellosis would be a welcome public health tool. Several *Shigella* vaccine candidates are under development [5–10]. Our findings indicate that vaccines to prevent shigellosis may need to be tailored according to prevalent species and serotypes, since only type-specific immunity has been demonstrated in humans [11–13] and cross-serotype protection is controversial [14].

We found an unexpectedly complex landscape of circulating *Shigella* strains in six Asian countries. The *Shigella* species believed to be dominant are S. flexneri in resource-poor countries and S. sonnei in industrialized nations. Consistent with previous reports [1], S. flexneri was most frequently isolated in the study sites in the five resource-poor countries (Bangladesh, China, Pakistan, Indonesia, and Vietnam), whereas in Thailand, which is rapidly becoming industrialized, S. sonnei was the most common species. Surprisingly, *S. boydii,* which had been thought to be relatively rare, was responsible for nearly one-quarter of shigellosis episodes at the Bangladesh site. Relatively few S. dysenteriae were detected during this surveillance project. Amongst the S. flexneri isolates were a surprisingly wide range of serotypes that varied across the Asian sites. The large variety in *Shigella* species and serotypes may explain the unusual age distribution of this enteric disease: Patients may remain susceptible to serotypes to which they have not been exposed earlier. The finding that S. sonnei is more frequently isolated in younger than in older children and S. flexneri is more frequently isolated from older than from younger shigellosis patients may be evidence for the steady replacement of *Shigella* strains with increasing age.

Not only do S. flexneri serotypes vary geographically, they vary temporally. We found statistically significant shifts in S. flexneri serotypes between observation years at each of the three sites where a comparison was possible. Temporal shifts in *Shigella* serotypes have been reported previously in Kolkata, India [43] but not in Santiago, Chile, where the serotype distribution was stable over prolonged periods [13]. Such shifts pose a double challenge for vaccine developers, who must choose the most relevant serotypes for inclusion in a multivalent vaccine, while knowing that replacement strains may emerge following the widespread introduction of the vaccine. To have a major epidemiological impact a shigellosis vaccine may need be a cocktail of antigens from several *Shigella* species and serotypes. An alternative approach would be to search for an antigen common to all *Shigella* species and serotypes. A vaccine that could elicit immunity against such a common antigen may be a promising future strategy to control shigellosis.

In conclusion, shigellosis is a frequent cause of diarrhea in the more impoverished areas of Asia. Although there were few medical complications associated with shigellosis, control of this disease could reduce of the overall diarrhea burden globally. The development of a vaccine protective against shigellosis is a highly desirable public health goal, but the development of such a vaccine is complicated by the variation in species and serogroups between sites, years, and age groups.

## Supporting Information

Alternative Language Abstract S1 Urdu Translation of the Abstract (1.2 MB PDF)

### Accession Numbers

The GenBank (http://www.ncbi.nlm.nih.gov) accession number of the gene discussed in this paper is *ipaH* (M32063).

## Abbreviation

CI: confidence interval
