# Supplementary material for: A Multicentre Study of Shigella Diarrhoea in Six Asian Countries: Disease Burden, Clinical Manifestations, and Microbiology
Source: PLoS Med. 2006 Sep 12;3(9):e353. doi: 10.1371/journal.pmed.0030353 (PMC1564174; doi:10.1371/journal.pmed.0030353)
Supplement: Supplementary file 1 [file pmed.0030353.sd001.pdf]

شگیلوسس سے بچاؤ کے لئے ایک کثیر الگرفتہ حفاظتی ویکسین کی ضرورت واہمیت

پس منظر:

پیچش یعنی شگیلوسس کی بیماری ترقی پذیر اور کم محدود ذرائع والے ممالک میں زیادہ پائی جاتی ہے۔ ایشیاء میں اس بیماری کی روک تھام میں رہنمائی کے لئے حال ہی میں کچھ اعداد و شمار حاصل ہوئے ہیں

طریقہءکار/بنیادی نتائج:

ہم نے مختلف ممالک یعنی بنگلہ دیش، چین، پاکستان، انڈونیشیا، ویت نام اور تھائی لینڈ میں مختلف مراکز میں عام آبادی پر مشتمل ہر عمر کے تقریباً 600,000 افراد میں اِس بیماری کی نوعیت، پھیلاؤ اور جراثیمی خصوصیات پر ایک تحقیق کی۔ اِس تحقیق کے نتیجہ میں سال 2002 اور 2004 کے درمیان دستوں کے 56,958 واقعات میں شگیلا جراثیم (یعنی پیچش پیدا کرنے والا جراثیم) 2,927 (5%) پائے گئے۔ علاج کے لئے آنے والے شگیلوسس میں مبتلا ہر عمر کے افراد کی شرح 2.1 فی 1000 فی سال اور 60 مہینوں سے کم عمر کے بچوں میں 13.2 فی 1000 فی تھی اور سوائے تھائی لینڈ کے تمام علاقوں میں شگیلا کی اقسام میں **ایس فلیکسنری قسم** (S.flexneri) سب سے زیادہ (68%; 1,976/2,927) تھی جبکہ تھائی لینڈ میں **ایس سونری قسم** (S.Sonneri) سب سے زیادہ پائی گئی تھی نیز **ایس فلیکسنری** قسم اور اِس کی ذیلی اِقسام کی شرحِ موجودگی مختلف ممالک میں مختلف یہاں تک کہ ہر سال مختلف پائی گئی۔

پی سی آر (PCR) ٹیسٹ (ایک طریقہ جس میں جراثیم کی جنیاتی ترکیب معلوم کی جاتی ہے) کے ذریعے ایسے پاخانے کے نمونے جن میں کسی بھی قسم کے جراثیم نہیں تھے، 33% ipaH (ایک جنیاتی حصہ) پایا گیا۔ حاصل ہونے والے نتائج میں شگیلا جراثیم کی قسم **ایس فلیکسنری** پر زیادہ تر عام اینٹی بائیوٹکس یعنی ایموکسیسیلن اور کوٹرائیموکسازول اثر پزیر نہیں تھیں (یہ جراثیم اِن ادویات سے مزاحم تھے)، یہاں تک کہ اینٹی بائیوٹک **سپروفلوکزاسن مزاحم ایس فلیکسنری** چائنا میں (18/305; 6%)، پاکستان میں (8/242; 3%)، اور ویتنام (5/282; 2%) میں پایا گیا۔

خلاصہ / اہمیت:

ماضی کے خیالات کے برعکس ایشیاء جیسے پسماندہ آبادی پر مشتمل خطہء میں شیگیلا جراثیم سب سے زیادہ پایا گیا ہے اِس لئے پیچش کی بیماری یعنی **شگیلوسس** سے روک تھام اور اِس سے بچاؤ کا بروقت فائدہ خطے میں دستوں کی بیماری کی شرح میں کمی کی صورت میں حاصل ہوسکتا ہے۔ پیچش سے متعلق یہ تحقیق اِس بات کی طرف اشارہ کرتی ہے کہ ایشیاء میں شگیلوسس کو روکنے کے لئے ایک ایسی فعال حفاظتی ویکسین کی ضرورت ہوگی جو شگیلا جراثیم کی تمام اِقسام اور ذیلی اِقسام کو پھیلنے سے روکنے اور پیچش کے علاج میں میں مددگار اور معاون ثابت ہو۔
